# Manipulations of Glutathione Metabolism Modulate IP_3_-Mediated Store-Operated Ca^2+^ Entry on Astroglioma Cell Line

**DOI:** 10.3389/fnagi.2021.785727

**Published:** 2021-12-17

**Authors:** Nawfel Mokrane, Yassin Snabi, Thierry Cens, Janique Guiramand, Pierre Charnet, Anaïs Bertaud, Claudine Menard, Matthieu Rousset, Marie-Céleste de Jesus Ferreira, Jean-Baptiste Thibaud, Catherine Cohen-Solal, Michel Vignes, Julien Roussel

**Affiliations:** ^1^UMR 5247 Institut des Biomolécules Max Mousseron (IBMM), Montpellier, France; ^2^Department of Biological Sciences, Université de Montpellier, Montpellier, France

**Keywords:** GSH (glutathione), store-operated calcium entry, sulforaphane, BSO (l-buthionine-sulfoximine), STIM and Orai, GSK 7975A, CRAC channel

## Abstract

The regulation of the redox status involves the activation of intracellular pathways as Nrf2 which provides hormetic adaptations against oxidative stress in response to environmental stimuli. In the brain, Nrf2 activation upregulates the formation of glutathione (GSH) which is the primary antioxidant system mainly produced by astrocytes. Astrocytes have also been shown to be themselves the target of oxidative stress. However, how changes in the redox status itself could impact the intracellular Ca^2+^ homeostasis in astrocytes is not known, although this could be of great help to understand the neuronal damage caused by oxidative stress. Indeed, intracellular Ca^2+^ changes in astrocytes are crucial for their regulatory actions on neuronal networks. We have manipulated GSH concentration in astroglioma cells with selective inhibitors and activators of the enzymes involved in the GSH cycle and analyzed how this could modify Ca^2+^ homeostasis. IP_3_-mediated store-operated calcium entry (SOCE), obtained after store depletion elicited by G_q_-linked purinergic P_2_Y receptors activation, are either sensitized or desensitized, following GSH depletion or increase, respectively. The desensitization may involve decreased expression of the proteins STIM2, Orai1, and Orai3 which support SOCE mechanism. The sensitization process revealed by exposing cells to oxidative stress likely involves the increase in the activity of Calcium Release-Activated Channels (CRAC) and/or in their membrane expression. In addition, we observe that GSH depletion drastically impacts P_2_Y receptor-mediated changes in membrane currents, as evidenced by large increases in Ca^2+^-dependent K^+^ currents. We conclude that changes in the redox status of astrocytes could dramatically modify Ca^2+^ responses to Gq-linked GPCR activation in both directions, by impacting store-dependent Ca^2+^-channels, and thus modify cellular excitability under purinergic stimulation.

## Introduction

Survival depends on the environment for the supply of oxygen, water and essential nutrients. Nevertheless, living organisms have to adapt continually their metabolic activity to environmental changes in order to keep themselves in homeostatic state. Indeed, environmental stresses such as caloric restriction, oxygen deprivation, exposure to pollutants, reactive oxygenated species (ROS) are sensed by various systems, so-called hormetic mechanisms, adapting cellular metabolic activity and oxygen consumption to specific conditions. These systems include the Nrf2 pathway which regulates redox homeostasis (Raefsky and Mattson, 2017) by promoting long-lasting cell protection against oxidative stress (Satoh et al., 2009; Robledinos-Antón et al., 2019) *via* the expression of enzymes involved in ROS scavenging. Some naturally occurring antioxidants as sulforaphane are potent inducers of this pathway (Murugaiyah and Mattson, 2015). More specifically, Nrf2 activation results in increased production of reduced glutathione (GSH) which is the prominent cellular antioxidant. GSH is a tripeptide resulting from the condensation of glutamate, cysteine and glycine (Lu, 2013). Its production is catalyzed by two enzymes, i.e., glutamate-cysteine ligase (GCL) and glutathione synthase (GS) which expressions are both upregulated by Nrf2 activation (Suzuki et al., 2013). Although hormetic mechanisms have been identified in the brain especially with regard to Nrf2 activation in astrocytes (Kraft et al., 2004), protective pathways are overwhelmed or inefficient in pathology and also during aging (Schmidlin et al., 2019; Yu and Xiao, 2021) to regulate oxidative stress. More specifically, the prominent role of Nrf2 pathway in oxidative stress handling throughout life is further emphasized by the fact that the inhibition of this pathway by progerin has been incriminated in the premature aging occurring during progeria disease (Kubben et al., 2016). The progressive reduction of Nrf2 activity during aging seems to involve not only changes in its availability but also from epigenetic regulations (Silva-Palacios et al., 2016). Indeed, the proteins involved in its turnover, including Keap 1, are also subjected to aging processes. In the CNS, deficits in GSH are associated with a wide array of pathologies including neurodegenerative diseases (Johnson et al., 2012; Gu et al., 2015; Singh et al., 2019) and neurodevelopmental ones (Bjørklund et al., 2020), as also observed in experimental models including rodent prenatal immune activation (Lanté et al., 2007). Decreased blood levels of GSH were evidenced in schizophrenic patients (Do et al., 2000; Lavoie et al., 2017). In this line, impeding GSH synthesis leads to the occurrence of behavioral and neurophysiological deficits in rodent models. Indeed, invalidating GCL gene results in increased oxidative stress and neuronal death, especially interneurons (Tosic et al., 2006; Kulak et al., 2013; Hardingham and Do, 2016).

In the brain, neuronal redox homeostasis is greatly dependent on astrocytic production of GSH (Dringen et al., 1999), as GSH or its metabolites can be shuttled to neurons by specific transporters. Indeed, the activity of the Nrf2 pathway is stronger in astrocytes than in neurons (Shih et al., 2003; Bolaños, 2016; Baxter and Hardingham, 2016). While astrocytes regulate neuronal ROS production under normal conditions, they can also produce ROS themselves when gaining a reactive phenotype under neuroinflammatory conditions (for review see Chen et al., 2020). In addition, reactive astrocytes seem to acquire specific intracellular Calcium regulation. For instance, Gq-linked metabotropic receptors responses are upregulated in reactive astrocytes as observed in neurodegenerative diseases models. This most likely impacts dramatically astrocytic functions such as gliotransmitter release and neuronal activity (Shigetomi et al., 2019).

These changes in astrocytic activity could be initiated by direct changes in redox balance. In this line, GSH depletion in glial cells, including astrocytes, leads to neuroinflammatory phenotype (Lee et al., 2010). Furthermore, a Ca^2+^-dependent glutamate release from microglial cells is observed upon hypoxia-mediated redox imbalance likely *via* the activation of IP_3_R which could participate in excitotoxic damages during brain insults (Socodato et al., 2018). Therefore, disrupted redox homeostasis in various glial cells could alter intracellular Ca^2+^ homeostasis and thus result in unregulated Ca^2+^-dependent release of factors as proinflammatory cytokines or glutamate endangering neuronal cell survival.

In the present work, we have thus evaluated the adaptations of Ca^2+^ homeostasis induced by changes in redox state. [GSH]_i_ was directly manipulated using either blockers of its synthesis, such as buthionine sulfoximine (BSO), or GSH cycle activators, such as sulforaphane (SFN) that enhances the expression of GSH synthetizing enzymes through the Nrf2 pathway activation. Experiments were carried out C6 astroglioma cells, a cellular model which recapitulates astrocytes features (Galland et al., 2019) and which exhibits Nrf2 pathway activation (Assis et al., 2014).

We found here that in C6 cells GSH depletion exacerbates store-operated calcium entry (SOCE) mechanism triggered by IP_3_-mediated Ca^2+^ release. By contrast, increasing GSH results in decreased SOCE. These changes are associated with genomic changes as boosting GSH concentration elicits decreased expression of the proteins involved in SOCE, i.e., the ER protein STIM2 and the membrane proteins Orai1 and Orai3 which form Ca^2+^ release-activated channels (CRAC).

## Materials and Methods

### Cell Cultures

Rat C6 glioma cells were a gift from Dr. Nathalie Chevallier (INSERM U1198). They were maintained in DMEM supplemented by 10% fetal calf serum in the presence of 100 U/mL penicillin and 100 μg/mL streptomycin.

### Measurement of Glutathione Content

Changes in cellular content of GSH were measured after monobromobimane (mBBr) labeling as previously described (De Jesus Ferreira et al., 2005). For this, C6 rat glioma cells were seeded in 96-well culture plates. When reaching 80% of confluence, cells were treated with BSO (10 μM) or SFN (10 μM) for 24 h. The culture medium was then replaced by extracellular medium containing 50 μM mBrB. Extracellular medium comprised: 124 mM NaCl, 3.5 mM KCl, 25 mM NaHCO3, 1.25 mM NaH_2_PO_4_, 1 mM CaCl2, 2 mM MgSO4, 10 mM D-glucose, and 10 mM HEPES (pH: 7.4). Incubation with mBBr lasted for 30 min then cells were washed with extracellular medium to remove unbound mBBr. Fluorescence was then measured *in cellulo* with a plate reader (Tecan “Spark” 20M) at 527 nm after excitation at 380 nm. Background fluorescence was obtained from mBBr unlabeled cells. For data processing, background fluorescence was subtracted to all fluorimetric signals which were further normalized to mBBr fluorescence recorded in control cells. An experimental determination was performed eight times per experiment. Data are expressed as percentages of at least three distinct experiments performed on different cell cultures.

### Measurement of Cell Viability

C6 rat glioma cells were seeded in 24-well culture plates. When reaching 80% of confluence cells were treated with either BSO (10 μM) or SFN (10 μM) or acivicin (100 μM) and returned to the incubator for 24 h. The oxidative stress was then induced by treating the cells with increasing concentrations of tert-butyl hydroperoxide (tBuOOH) for 24 h. When tested, the intracellular Ca^2+^ chelator 1,2-Bis(2-aminophenoxy)ethane-N,N,N′,N′-tetraacetic acid tetrakis(acetoxymethyl ester) (BAPTA-AM 1–10 μM) was added 1 h prior to the tBuOOH application. Cell viability was measured by assaying mitochondrial activity using MTT (3-[4,5-dimethylthiazol-2-yl]-2,5-diphenyl-tetrazolium bromide) transformation, as previously described (De Jesus Ferreira et al., 2005). Data are presented as percentages of MTT transformation normalized to basal obtained in cells without any treatment. They are means (±S.E.M.) of at least three individual determinations performed on different cell cultures; each determination being performed in triplicate per experiment.

### Measurement of Intracellular Ca^2+^ Concentration

Intracellular calcium concentration ([Ca^2+^]_i_) was measured using the fluorescent indicator fura-2. For this purpose, cells grown on glass coverslips were loaded with fura-2 by a 30 min incubation at 37 °C with 1 μM fura-2-AM and 0.02% Pluronic in the extracellular solution described above. [Ca^2+^]_i_ was monitored by videomicroscopy. After rinsing, the glass coverslip was transferred to the recording chamber mounted on an inverted microscope (Leica, DMIRB), continuously superfused with extracellular medium. Fura-2 emission was obtained by exciting alternatively at 340 and 380 nm with a rotating filter wheel (Sutter Instruments) and by monitoring emissions (F340 and F380) at 510 nm. Fluorescent signals were collected with a CCD camera (Hamamatsu), digitized, and analyzed with image analysis software (Acquacosmos, Hamamatsu). The ratio of emissions at 510 nm (F340/F380) was recorded in cells every second. Experiments were carried out at room temperature and drug application was performed with a gravity-fed system. Data are expressed as averages (±SEM) of the ratio between the fura-2 fluorescence values of 340/380 nm excitation wavelengths ratios (F340/F380) normalized to the corresponding basal F340/F380 measured prior to any drug application. When required, F340/F380 ratio was calibrated into [Ca^2+^]_i_ (nM) according the following equation (Grynkiewicz et al., 1985):


$$\left[C\,a^{2+}\right]\,i=K\,d.\frac{\left(R-R\,m\,i\,n\right)}{\left(R\,m\,a\,x-R\right)}.\left(\frac{S\,2\,f}{S\,2\,b}\right)$$

With Kd = 224 nM, R = F340/F380, S2f = F380 without Ca^2+^ and S2b = F380 with saturating Ca^2+^.

R_max_ was obtained by applying the Ca^2+^ ionophore 4Br-A23187 (5 μM). R_min_ was then recorded by substituting the extracellular medium for Ca^2+^-free extracellular medium with no added Ca^2+^ and supplemented with EGTA (2 mM).

Graphs presenting time-courses of F340/F380 ratio changes have been obtained by averaging data from a population of cells recorded individually during one single representative experiment. When required, the Areas Under Curve (AUC) of the Ca^2+^ entries elicited by tBuOOH application or by store depletion (SOCE) were calculated by integrating F340/F380 ratio over 5 min starting at the time of Ca^2+^ reinstatement in the extracellular medium. In graphs of pooled data, “n” values represent the entire population of cells recorded from at least three independent cultures (N).

### Electrophysiology

Membrane currents were recorded using whole-cell patch-clamp method. Cells grown on glass coverslips were transferred to a recording chamber of an upright microscope, continuously superfused with the extracellular medium. Experiments were performed at room temperature with glass pipettes (4–5 MΩ resistance) filled with the intracellular solution comprising 140 mM KGluconate, 4 mM NaCl, 1 mM MgCl_2_, 1 mM EGTA, 5 mM HEPES, 2 mM MgATP, 0.6 mM NaGTP, pH = 7.4 (KOH). Cells were held at 0 mV and membrane currents were recorded by applying voltage ramps from −100 to 100 mV every 10 s. Access resistance was monitored by applying a 10 mV voltage step at the end of the voltage ramp. Currents were collected and amplified with an Axoclamp 200B amplifier (Molecular Devices) and digitized (Digidata 1322, Molecular Devices). They were analyzed with John Dempster’s software WCP. Drugs were applied at the desired concentration *via* a gravity-fed application system. Data are presented as means ± S.E.M. on graphs plotting pooled data.

### Two-Step Real-Time RT PCR

Total RNA extraction was performed after cell collection using TRI Reagent^®^ (Molecular Research Center, Inc.) according to the manufacturer’s instructions. Final concentration of 1 ng/mL total RNA was reverse-transcribed to first-strand cDNA using oligo-dT primer and SuperScript*™* II Reverse Transcriptase (Invitrogen*™*) as described by the manufacturer. Real-time PCR was then carried out using SensiFAST*™* SYBR^®^ No-ROX Kit (Bioline) in LightCycler^®^ 480 Instrument II (Roche Molecular Systems, Inc.). The primers used for the amplification of target cDNAs are shown in Table 1. They were designed with IDTDNA Technologies software. The access numbers for the target genes were obtained from NCBI data bank. All reactions were performed in a 1.5 μL volume using the following PCR program: initial denaturation at 95°C for 2 min, followed by 45 tri-step cycles of amplification: 10 s at 95°C, 10 s at 65°C, and 10 s at 72°C. Negative controls were obtained by running PCR with H_2_O instead of the template. Specificity was verified by melting curve analyses and agarose gel electrophoresis of the amplicon. Levels of specific transcripts were given by the threshold cycle (Ct) values and the data were normalized for the corresponding β-actin RNA contents.

**TABLE 1:** Oligonucleotide sequences of the primers used for real-time PCR to assess the expression the genes of interest.

Gene name	Forward 5′→3′	Reverse 3′→5′
β-actin	TCACTATCGGCAATGAGCG	GGCATAGAGGTCTTTACGGATG
HO-1	CCTGCTAGCCTGGTTCAAG	CATAAATTCCCACTGCCACG
Nrf2	GCTATTTTCCATTCCCGAGTTAC	ATTGCTGTCCATCTCTGTCAG
GCLC	TTCCTACATTCCACTGTCCAAG	CCTTGCTACACCCATCCAC
TRPM7	TGCCTGTAAAATCT ATCGTTCAATG	TCCAGTAATTCAACGGCGAG
TRPV1	CCCTTTATGACCTGTCCTGC	AAATCTGTCCCACTTGTCCTG
TRPC1	AGGTGACTTGAAC ATAAATTGCG	GTTGAGTATTCCGG ATTCTGAATTC
Orai1	TGAAGTTCTTACCGCTCAAGAG	CATGATGGCAGTGGAGGC
Orai3	TGTGGGACTAGTGTTTATGGC	AACAGTCTAAAGCTGGGCTC
STIM1	CGCGCTCAACATAGATCCCA	TGGGGGACTGCATGGACAA
STIM2	TGCTCTTCGGGCTGTTGGT	TACAGGGATCTGTCAGCAGCG

### Western Blotting

Cells were washed with PBS and lysed using RIPA Lysis Buffer (Boster) containing 1% NP-40, 0.5% sodium deoxycholate and 0.1% sodium dodecyl sulfate (SDS). Samples were then boiled for 5 min in a Laemmli Sample Buffer (BioRad) and loaded on a 12% SDS –polyacrylamide gel (6–8 mg/mL). After electrophoresis, proteins were electro-transferred (100 mV, 1 h) to nitrocellulose membrane using a sandwich blotting system. Blots were first incubated with 5% BSA and 0.1% Tween in Tris-buffered saline (TBS) for 1 h at room temperature, and then with either polyclonal rabbit primary antibody against Orai1 (1:1,000, rabbit Sigma-Aldrich, O8264), STIM2 (1:2,000, rabbit Sigma-Aldrich, S8572) or monoclonal mouse actin (1:100, mouse Sigma-Aldrich, A2066) at 4°C overnight. The appropriate horseradish peroxidase-conjugated secondary antibody IgG was added at a dilution of 1:10,000 for 1 h at RT. The peroxidase was detected with ECL Western Blotting Reagent (Amersham).

### Statistical Analyses

Statistical analyses were performed using SigmaStat software (Systat software Inc.). Data were first tested for normality and equality of the variances. In case of one or the other test failure (*p* < 0.05) then non-parametric methods, mainly ANOVA on rank, were used for comparisons. If both tests passed, then parametric methods were used. Specific tests used for each experiment were described in figure legends. Data obeying normality were represented as mean ± SEM; in other case data were represented as median, deciles and quartiles.

## Results

### Modulation of Glutathione Metabolism Alters Cell Resistance to Oxidative Stress Toxicity Elicited by tBuOOH

**FIGURE 1:**
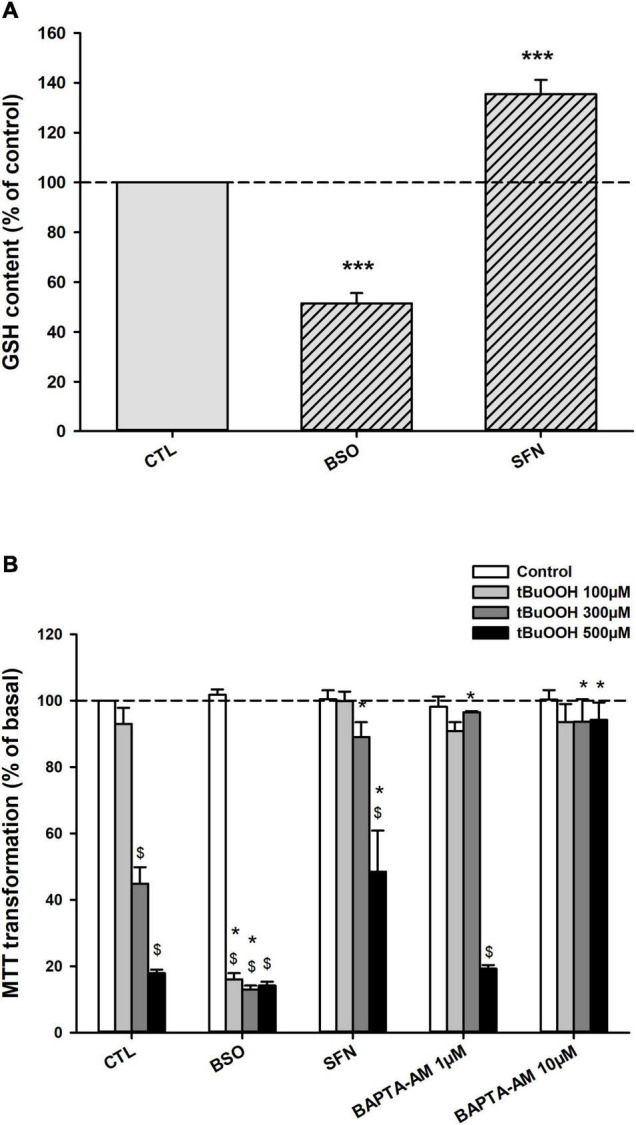
Glutathione content and C6 glioma cell survival under oxidative stress after manipulating GSH metabolism. **(A)** [GSH]_i_ content in control, BSO- and SFN- pretreated cells. GSH was measured *in cellulo* with mBBr 24H following treatments. On the graph, data are presented as percentages of mBBr fluorescence intensity normalized to basal mBBr fluorescence measured in control untreated cells. ***p < 0.001 when comparing GSH basal level with either treatment (one-sample *t*-test). **(B)** Cell survival of control, BSO-, SFN-, or BAPTA-AM pretreated cells challenged by increasing concentrations of tBuOOH (100, 300, and 500 μM). Cells were pretreated for 24H with 10 μM BSO or 10 μM SFN, or for 1 h with BAPTA-AM (1 or 10 μM) before the exposure to tBuOOH. Cell survival was assayed 24 h later, by measuring MTT transformation. Data are presented as percentages of basal MTT transformation in control untreated cells. *p < 0.05 vs. respective tBuOOH in untreated cells, ^$^*p* < 0.05 vs. respective control in the absence of tBuOOH (two-way ANOVA followed by Holm-Sidak *post-hoc* test for multiple comparisons).

In order to modulate GSH levels, cells were pretreated for 24 h with either buthionine sulfoximine (BSO) or sulforaphane (SFN). The intracellular GSH concentration ([GSH]_i_) was assayed *in cellulo* by labeling living cells with monobromobimane (mBBr). In cells exposed to 10 μM BSO, a γ-glutamyl-cysteine ligase (GCL) blocker, mBBr fluorescence was significantly reduced to 51 ± 4% (*n* = 11) as compared to untreated cells. Treatment with 20 μM BSO did not further reduce GSH content. On the opposite, mBBr fluorescence was increased to 135 ± 6% (*n* = 7) of control fluorescence in cells treated with 10 μM SFN, a strong activator of Nrf-2 pathway (Figure 1A).

We next verified whether [GSH]_i_ modulation effectively altered cell sensitivity toward oxidative stress. For this, the toxicity of tert-butyl hydroperoxide (tBuOOH) was measured on C6 cell pretreated or not with BSO or SFN (Figure 1B). Under control conditions, a 100 μM tBuOOH exposure led to little or no cell damage (93 ± 5% of cell survival, *n* = 12). By contrast, when applied at 500 μM, a strong cell death was observed (18 ± 1% of cell survival, *n* = 12). Treatment with 300 μM tBuOOH, resulted in moderate cell death survival (45 ± 5% *n* = 12 of cell survival). Thus, analyzing changes in cell survival profile obtained after tBuOOH treatments at 100, 300, and 500 μM enabled to measure both sensitization and resistance to oxidative stress.

Neither SFN, nor BSO pretreatment affected cell survival under basal conditions. Cell survival was 101 ± 3% and 102 ± 2% (*n* = 8) in SFN and BSO pretreated cells, respectively (Figure 1B). Increasing [GSH]_i_ with SFN pretreatment protected cells against peroxide toxicity: upon 500 μM tBuOOH, cell survival was 49 ± 12% (*n* = 8) in cells treated with SFN, as compared to 18 ± 3% obtained in control cells (Figure 1B). Other agents triggering Nrf2 pathway as dimethyl fumarate and tertio butyl hydroquinone exhibited similar effects as SFN (data not shown). In contrast, decreasing [GSH]i with BSO sensitized cells to peroxide toxicity. Indeed, in the presence of 100 μM tBuOOH, a concentration which did not induce any significant cell death in control cells, cell survival was reduced to 16 ± 2% after BSO pretreatment (Figure 1B).

To establish the putative link between oxidative stress and calcium homeostasis disruption in C6 glioma cells, intracellular Ca^2+^ variations were buffered using the cell permeant calcium chelator, BAPTA-AM. The treatment with 1–10 μM BAPTA-AM of C6 cells prevented cell death induced by the application of tBuOOH (100–500 μM) (Figure 1B). Cell survival was 94 ± 5% (*n* = 5) after exposing 10 μM BAPTA-loaded cells to 500 μM tBuOOH. This strengthened the requirement of intracellular calcium increases for the expression of peroxide-mediated toxicity in C6 cells.

### Modulation of Glutathione Metabolism Alters Oxidative Stress-Induced [Ca^2+^]_i_ Changes

**FIGURE 2:**
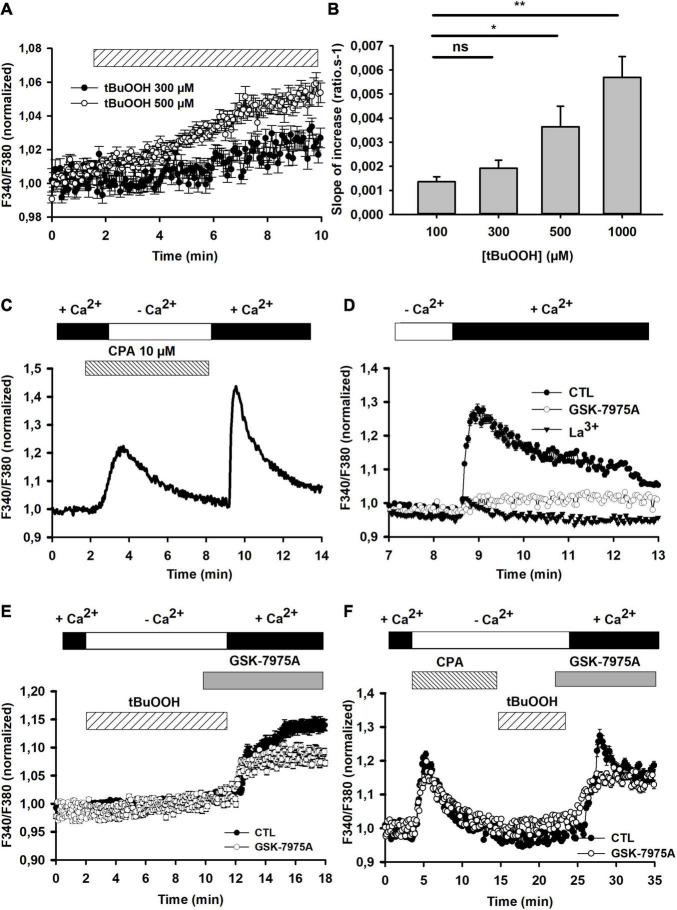
[Ca^2+^]i changes in the presence of tBuOOH. **(A)** Pooled data illustrating [Ca^2+^]_i_ changes induced by 300 and 500 μM tBuOOH (*n* = 20 and 21 cells, respectively). These curves were fitted with a linear regression. The slope values were calculated using linear regression function on SigmaPlot Software from the onset of F340/F380 increase for 10 min (*R*^2^ = 0.9416 for F340/F380 change induced by 500 μM and *R*^2^ = 0.6598 for F340/F380 change induced by 300 μM). **(B)** Concentration-dependent changes of the slope of tBuOOH-induced [Ca^2+^] increase. Data are expressed as averages of the slopes of F340/F380 ratios time-courses obtained in 47, 97, 100, and 50 cells for the applications of 100, 300, 500, and 1,000 μM, respectively. “ns” or *p < 0.05 or **p < 0.01 when comparing slopes to the slope obtained in the presence of 100 μM tBuOOH (one way ANOVA followed by Holm Sidak *t*-test). **(C)** Illustrative recording of store-operated capacitive Ca^2+^ entry (SOCE) elicited by cyclopiazonic acid (CPA, 10 μM) in C6 glioma cells. Cells were challenged by CPA in Ca^2+^-free medium (including 2 mM EGTA) for 10 min before [Ca^2+^]_e_ was restored. **(D)** Pharmacological characterization of SOCE. Following CPA stimulation in the absence of extracellular Ca^2+^, CRAC blockers, i.e., GSK-7975A (5 μM) or La^3+^ (100 μM) were applied 2 min prior to reinstating extracellular calcium concentration at 2 mM to reveal SOCE. **(E)** Assessment of Ca^2+^ release from internal stores by tBuOOH exposure. Cells were challenged by tBuOOH (500 μM) in Ca^2+^-free medium (including 2 mM EGTA) for 10 min before [Ca^2+^]_e_ was restored. When required, the CRAC blocker was applied 2 min prior to [Ca^2+^]_e_ reinstatement. **(F)** As compared to the experiment in panel **(E)**, in this case the tBuOOH exposure was preceded by the application of CPA to empty internal Ca^2+^ stores. The graphs in E and F plot averaged data obtained from 17 (control) and 29 (+GSK-7975A) individual cells and from 22 (control) and 20 (+GSK-7975A) individual cells, respectively.

We next evaluated how [GSH]_i_ manipulation impacted oxidative stress-mediated [Ca^2+^]_i_ changes. With this aim, the effect of tBuOOH was first examined on control cells. This peroxide induced a concentration-dependent increase in [Ca^2+^]_i_ in C6 cells which was linear for the first 10 min after applying it (Figures 2A,B). Cytoplasmic Ca^2+^ variation may result from influxes from the extracellular medium to cytoplasm or/and from Ca^2+^ release from internal stores including endoplasmic reticulum (ER). In extracellular Ca^2+^-depleted medium (Figure 2E) or in the presence of 100 μM lanthanum ions (La^3+^), a non-selective calcium channel blocker, tBuOOH-induced [Ca^2+^]_i_ accumulation was completely inhibited (not shown), suggesting the occurrence of Ca^2+^ entries under oxidative conditions.

**FIGURE 3:**
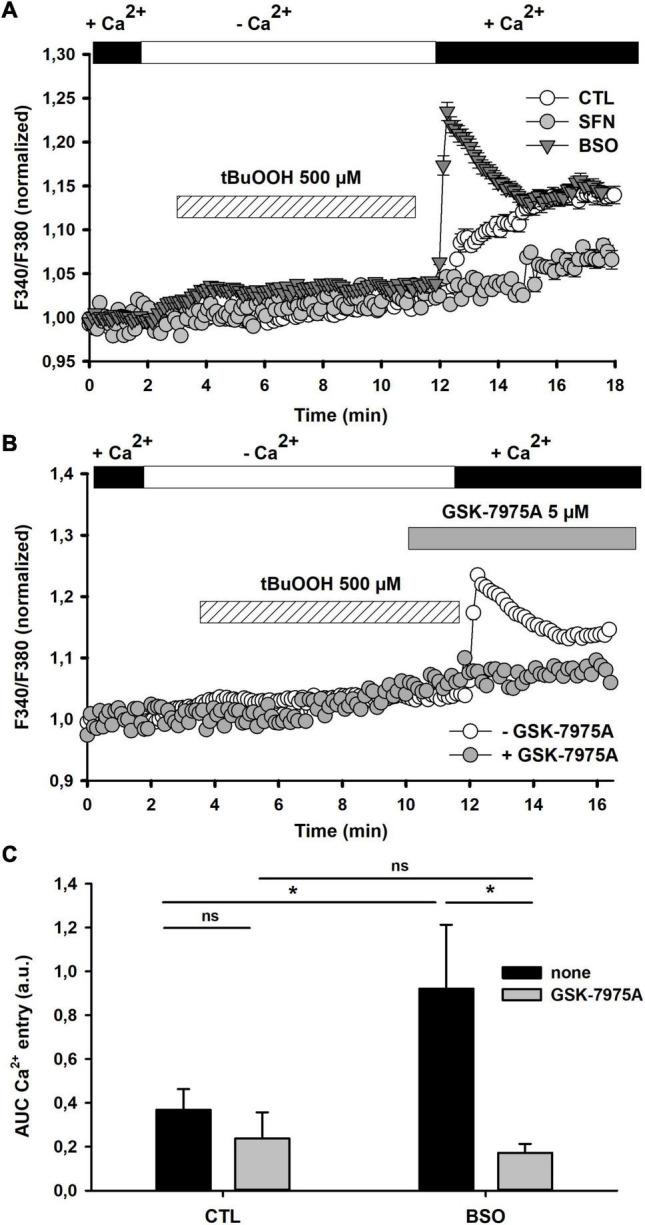
Effect of [GSH]_i_ manipulations on tBuOOH-mediated [Ca^2+^]_i_ changes. **(A)** [Ca^2+^]_i_ changes elicited by tBuOOH (500 μM) in control, SFN- and BSO-pretreated cells. The graph represents an average of the [Ca^2+^]i changes measured in 28, 17, and 19 individual cells for control, SFN- and BSO-pretreated cells, respectively. **(B)** Effect of GSK-7975A on the tBuOOH-induced [Ca^2+^]_i_ increase in BSO-pretreated cells. The graphs have been generated by averaging [Ca^2+^]i changes measured in BSO-pretreated individual cells recorded in the absence (*n* = 29) or in the presence of GSK-7975A (*n* = 29). The CRAC blocker was applied 2 min prior to the [Ca^2+^]_e_ reinstatement. **(C)** Recapitulative graphs plotting F340/F380 ratio changes elicited by tBuOOH (500 μM) in control and BSO-pretreated cells and in the presence of GSK-7975A. Data are means ± SEM of AUC measured in 86 cells (untreated control cells), 123 cells (untreated with GSK-7975A), 61 cells (BSO-pretreated), and 66 cells (BSO-pretreated with GSK-7975A). *p < 0.05 vs. respective control in the absence of GSK-7975A (two-way ANOVA followed by Holm-Sidak *post-hoc* test for multiple comparisons).

The contribution of intracellular calcium store release to the [Ca^2+^]_i_ increase elicited by tBuOOH and the potential induction of store-operated capacitive calcium entries (SOCE) as a source of Ca^2+^ influx was further evaluated. First, the occurrence of SOCE was examined in C6 cells thanks to their sensitivity to the selective CRAC blocker, GSK-7975A. SOCE were triggered by depleting Ca^2+^ from the ER with cyclopiazonic acid (10 μM, CPA), a reversible SERCA (Sarcoplasmic and Endoplasmic Reticulum Ca^2+^ ATPase) pump blocker, in Ca^2+^-free medium, and revealed by reinstating extracellular calcium concentration at 2 mM (Figure 2C). As expected, the addition of CPA induced a transient increase in Ca^2+^ in the absence of extracellular calcium, reflecting the release from ER; then reinstating extracellular calcium at 2 mM induced a large increase in intracellular Ca2+, reflecting the SOCE (Figure 2C). Consistently, this Ca^2+^ increase was blocked by 5 μM GSK-7975A or by 100 μM La^3+^ ions (Figure 2D). The AUC for the SOCE plotted in the Figure 2D were 0.67 ± 0.02 in control and 0.21 ± 0.06 in the presence of GK-7975A (*n* = 30 each). Then, the potential induction of SOCE by tBuOOH was evaluated. For this, tBuOOH was transiently applied in the absence of extracellular Ca^2+^. Under these conditions, no Ca^2+^ rise could be evidenced during the application of tBuOOH, but a large Ca^2+^ increase appeared when extracellular calcium was restored. This increase was partially blocked by GSK-7975A (Figures 2E, 3C). Thus, in C6 cells under control conditions tBuOOH did not induce any measurable intracellular calcium release, but evoked an entry of calcium upon extracellular calcium reinstatement partially sensitive to GSK-7975A. To confirm this entry was not a SOCE, intracellular Ca^2+^ stores were first depleted by CPA prior to the tBuOOH application. Under these conditions, the Ca^2+^ entry was still observed and retained its partial sensitivity to GSK-7975A (Figure 2F). The AUC for the Ca^2+^ entry plotted in the Figure 2F were 1.64 ± 0.34 in control and 1.04 ± 0.30 in the presence of GK-7975A (*n* = 20 each). The La^3+^-sensitive Ca^2+^ entry triggered by tBuOOH is thus largely independent of SOCE mechanism under control conditions.

We next evaluated how changing GSH content altered [Ca^2+^]_i_ changes elicited by tBuOOH. First of all, resting [Ca^2+^]_i_ was measured in control, BSO- and SFN-pretreated cells. It appears that [GSH]_i_ manipulations did not significantly alter basal [Ca^2+^]_i_ in these three groups of cells: resting [Ca^2+^]_i_ was 65 ± 2 nM, 66 ± 2 nM, 72 ± 5 nM, respectively (see Table 2). In SFN-pretreated cells, tBuOOH exposure elicited a Ca^2+^ entry lower than in control cells. By contrast, in GSH-depleted cells with BSO, a significant potentiation of the tBuOOH-evoked [Ca^2+^]_i_ increase was observed (Figures 3A,C). Moreover this potentiation [Ca^2+^]_i_ was almost completely inhibited by CRAC blocker (Figures 3B,C). Thus, GSH-depletion apparently sensitized CRAC to induce Ca^2+^ entry.

**TABLE 2:** Evaluation of SFN- or BSO-pretreatments on basal [Ca^2+^]i and P_2_YR response elicited by ADP applications.

	Control	SFN	BSO
Basal [Ca^2+^]_i_ (nM)	65 ± 2 (*n* = 81)	72 ± 5 (*n* = 64)	66 ± 2 (*n* = 76)
EC_50_ (μM)	0.3 ± 0.1	1 ± 0.2*	0.05 ± 0.01
nH	1.3 ± 0.04	1.1 ± 0.24	1.1 ± 0.06
ADP-induced [Ca^2+^]_i_ peak (μM)	4.5 ± 0.53 (*n* = 101)	0.5 ± 0.05* (*n* = 79)	5.6 ± 0.83 (*n* = 80)

*EC_50_ and Hill number (nH) were calculated from data obtained in three independent cultures. “n” applies to the total number of cells recorded to calculate ADP-mediated peak [Ca^2+^]_i_. Data are means ± SEM.*

*p < 0.05 when comparing to control cells.

### Impacts of Manipulating Glutathione Content on IP_3_-Mediated Store-Operated Calcium Entry

**FIGURE 4:**
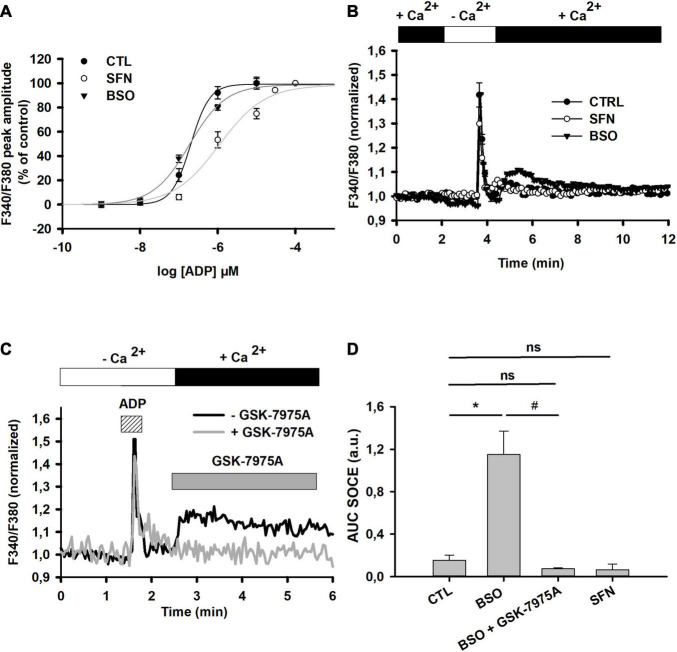
Effect of SFN and BSO treatments on P_2_Y receptors-mediated SOCE. **(A)** Concentration-dependent changes of P_2_Y receptor-mediated [Ca^2+^]_i_ transient increase in control, SFN- and BSO-pretreated cells. P_2_Y receptors were stimulated by increasing concentrations of ADP (1, 10, 100 nM and 1, 10 μM) in the absence of extracellular calcium and the peak amplitude was determined. Curves were generated after fitting data with sigmoidal function (Sigmaplot). Such fittings allowed to determine EC50, nH, and Emax in each individual dose response curves, which are reported in Table 2. **(B)** SOCE triggered by ADP (10 μM) in control, SFN- and BSO-pretreated cells. The graphs were generated by averaging data from 46 (control), 32 (SFN-pretreated), and 35 (BSO-pretreated) individual cells, respectively. **(C)** Single example depicting the effect of GSK-7975A on ADP-induced SOCE in BSO-pre-treated cells. CRAC blocker was applied at 5 μM 30 s prior to [Ca^2+^]_e_ reinstatement. **(D)** Recapitulative graph plotting the AUC of the SOCE elicited by ADP (10 μM) in control, SFN- and BSO-pre-treated cells and in the presence of GSK-7975A (5 μM). AUC were calculated by integrating F340/F380 normalized changes over 5 min after reinstating Ca^2+^ in the extracellular medium. Data are means ± SEM of AUC measured in 72 cells (*N* = 3; untreated control cells), 55 cells (*N* = 3; BSO-pretreated), 30 cells (*N* = 3; BSO-pretreated with GSK), and 77 cells (*N* = 3; SFN-pretreated). *p < 0.05 when comparing with control cells and # when comparing with BSO-treated cells without GSK-7975A (two Way ANOVA followed by Holm Sidak post-hoc test for multiple comparisons).

The sensitization by GSH depletion of CRAC observed under oxidative stress suggested that SOCE elicited by Ca^2+^ mobilization from the ER should be modulated by GSH content manipulation (Figure 3). We have thus tested whether the activation of the IP_3_/Ca^2+^ mobilization pathway exhibited sensitivity to the GSH concentration. The stimulation of G_*q*_-linked metabotropic P_2_Y receptors with ADP elicited intracellular mobilization from IP_3_-sensitive internal calcium stores in C6 cells (Figures 4A,B). Pretreating cells with BSO or SFN significantly impacted IP_3_-mediated transient Ca^2+^ increase as shown by the comparisons of ADP concentration-response curves. As compared to control cells, the EC50 of ADP on calcium transient was slightly but non-significantly reduced in BSO-treated cells. By contrast, the EC50 of ADP effect on calcium transient was significantly increased in SFN-treated cells (1 ± 0.2 μM), while Hill number remained unchanged. Moreover, the maximal effect of ADP seemed to be similar in the different conditions (Table 2).

We have then evaluated whether store-operated calcium entry (SOCE) following P_2_Y receptor activation was altered by [GSH]_i_ manipulations. Following extracellular calcium reinstatement ADP triggered a significant calcium influx in BSO-pretreated cells (Figures 4B,D). This influx was blocked by GSK-7975A (Figures 4C,D). SOCE was barely detected in either control or SFN-treated cells. Thus, in C6 glioma cells [GSH]_i_ depletion greatly potentiated SOCE triggered by IP_3_-mediated responses.

Since both ADP evoked calcium transients and SOCE could be modified following intracellular GSH content manipulation, we have then examined the impact of BSO or SFN pretreatments on the capacity of cells to accumulate Ca^2+^ within the ER and on SOCE occurrence using CPA (Figure 2C). The area under the curve (AUC) of both Ca^2+^ mobilization and Ca^2+^ entry did not exhibit significant differences whatever the treatment applied (Table 3). Therefore, GSH manipulation neither modified Ca^2+^ load within the ER nor SOCE elicited by SERCA inhibition. This suggested that GSH metabolism modulation predominantly affected IP_3_ receptor-mediated SOCE.

**TABLE 3:** Evaluation of SFN- or BSO-pretreatments on basal [Ca^2+^]_i_ and SOCE parameters, i.e., AUC calculated from Ca^2+^ increase in the presence CPA and AUC of Ca^2+^ increase obtained after restoring extracellular Ca^2+^ concentration to 1 mM.

	Control	SFN	BSO
CPA (AUC)	0.59 ± 0.01 (*n* = 82)	0.65 ± 0.02 (*n* = 63)	0.65 ± 0.01 (*n* = 83)
SOCE (AUC)	0.67 ± 0.02 (*n* = 55)	0.62 ± 0.01 (*n* = 63)	0.61 ± 0.02 (*n* = 81)

*“n” applies to the total number of cells recorded. Data are means ± SEM.*

### Impacts of Manipulating Glutathione Content on STIM and Orai Expressions

The effects of GSH content manipulations on C6 glioma cells, were then studied on the expression of STIM and Orai, two interacting proteins responsible for SOCE after ER-Ca^2+^ store release.

**FIGURE 5:**
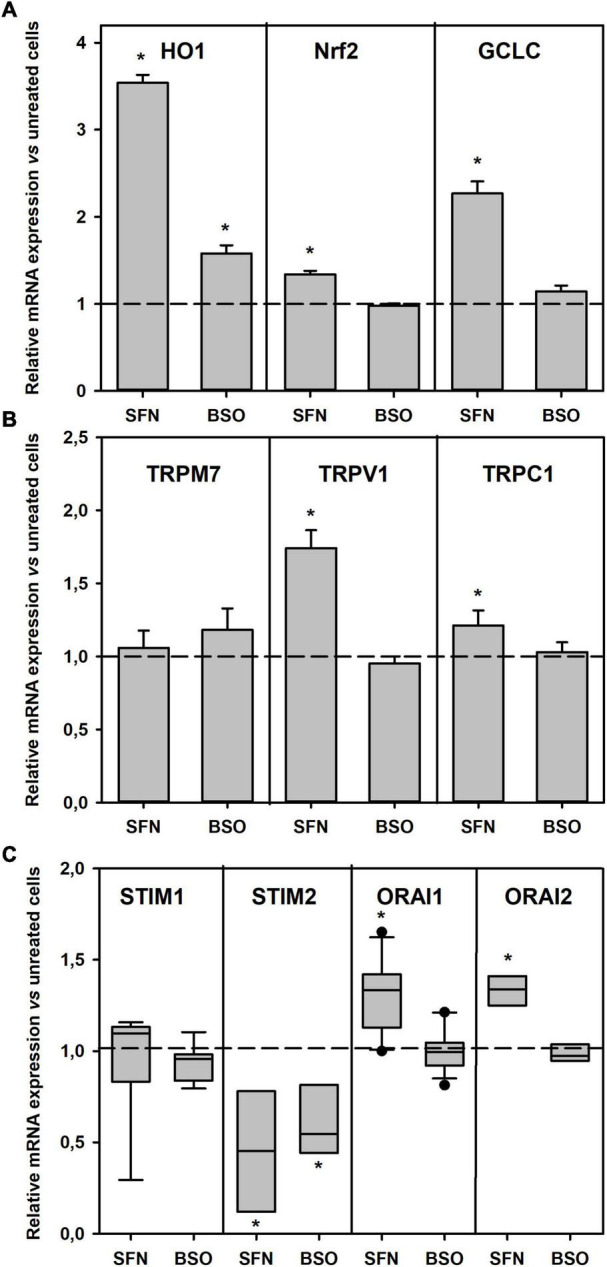
Variations of gene expression following GSH metabolic manipulations. mRNA expressions of the genes of interest were normalized to the mRNA expression of the β-actin gene obtained in control, BSO- and SFN-treated cells. **(A)** mRNA expressions of Nrf2, Heme Oxygenase 1 (HO-1), and glutamate cysteine ligase catalytic subunit (GCLC) genes. **(B)** mRNA expressions of oxidization sensitive TRP channels genes, including TRPV1, TRPC1, and TRPM7. In A and B, *p < 0.05 vs. control cells (one-way ANOVA on repeated measures followed by Dunnett’s *post-hoc* test). **(C)** mRNA expressions of SOCE proteins genes, including STIM1, STIM2, Orai1, and Orai3. Specific mRNAs were quantified by qRT-PCR. *p < 0.05 vs. control cells (ANOVA on rank on repeated measures followed by Dunnett’s *post-hoc* test).

First, mRNAs of NRF2, as well as of Heme Oxigenase 1 (HO-1) and GCL, two enzymes whose expression is known to be under the control of NRF2, were quantified by qRT-PCR (Figure 5A). Significant increases in HO-1, Nrf2, and GCLC (the catalytic subunit of GCL) mRNA expressions were evidenced in SFN treated cells. Treatments with BSO had no effect on NRF2 and CGLC mRNA expression, but slightly increased the expression of HO-1 mRNA.

The mRNA expressions of the oxidative stress-sensitive TRP channels were examined. The TRPM2 mRNA was not detectable in these cells (not shown). BSO pretreatment had no effect on the expression of TRPM7, TRPV1, and TRPC1 mRNAs, while SFN pretreatment significantly increased the TRPV1 mRNA expression, and to a lesser extent that of TRPC1 (Figure 5B).

Finally, mRNA expressions of the proteins involved in SOCE, i.e., STIM and Orai, were measured. SFN pretreatment induced a significant decrease in STIM2 mRNAs accompanied by significant increases in Orai1 and Orai3 mRNA expressions. BSO pretreatment significantly diminished STIM2 mRNA expression (Figure 5C).

**FIGURE 6:**
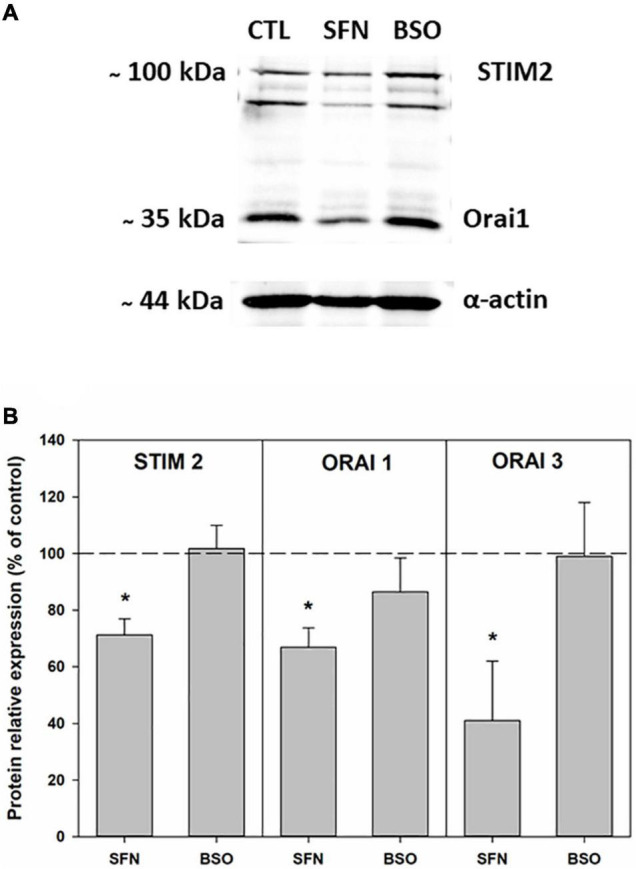
STIM2, Orai1, and Orai3 proteins expressions in control, SFN- and BSO-pretreated cells. **(A)** Illustrative Western blots depicting STIM2 and Orai1 proteins detection. **(B)** Pooled data of the expressions of STIM2 proteins (*n* = 5 for each condition) and of Orai1 proteins (*n* = 6 for each condition) and of Orai3 proteins (*n* = 3 for each condition). On the graphs data are expressed as averages of relative expressions obtained by normalizing STIM2 or Orai1 or Orai3 proteins expressions to β-actin expression. *p < 0.05 vs. control cells (one tailed *t*-test).

SOCE protein expressions were further examined by western blot. We observed that SFN treatment led to a significant decrease in both STIM2, Orai1, and Orai3 expressions (Figures 6A,B). The decreased expression of STIM and Orai proteins could explain the diminished SOCE mediated by IP_3_/Ca^2+^ mobilization observed in these cells after this treatment.

### Impacts of Manipulating Glutathione Content on ADP-Mediated Currents

**FIGURE 7:**
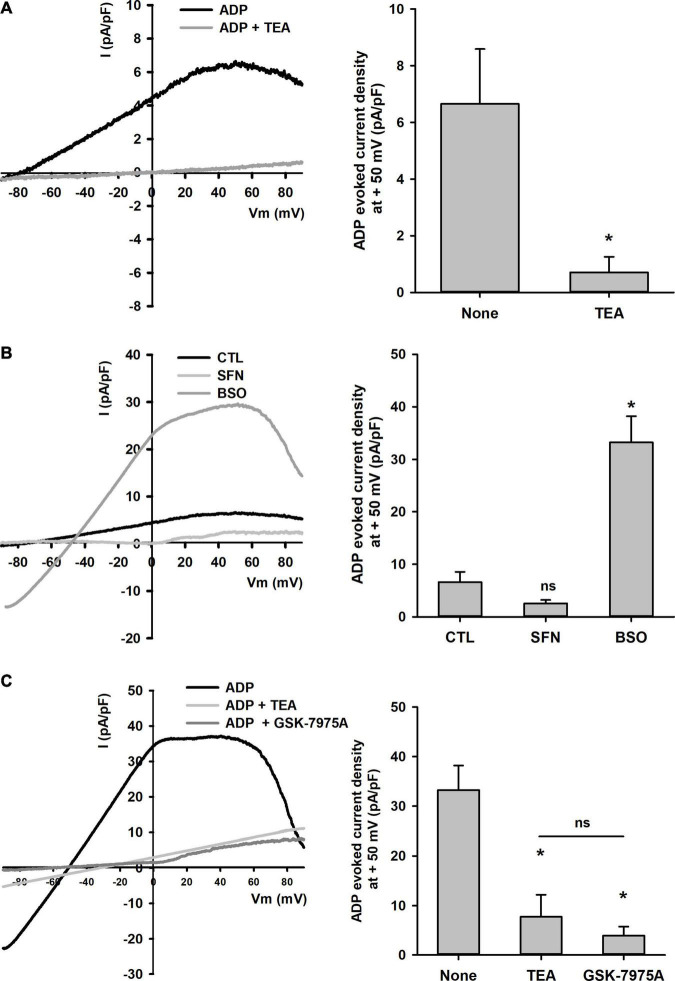
Effects of SFN and BSO treatments on P_2_Y receptors-mediated membrane currents. **(A)** On the left, single traces of currents recorded after ADP (10 μM) application on control cells either in the absence or in the presence of TEA (20 mM) are shown. Currents were recorded by applying voltage ramps from -100 to 100 mV every 10 s. On the right, recapitulative histograms plot P_2_Y receptor-mediated current densities measured at +50 mV in these cells (*n* = 4). *p < 0.05 vs. control cells untreated cells (Student’s *t*-test). **(B)** On the left, sample traces of currents recorded after ADP (10 μM) application on control, BSO- and SFN-pretreated cells are shown. On the right, recapitulative histograms plot P_2_Y receptor-mediated current densities measured at +50 mV in these cells (*n* = 4, 5, and 3, for control, SFN, and BSO, respectively). *p < 0.05 vs. control cells (ANOVA followed by Dunnett’s *post-hoc* test). **(C)** Blockade of ADP-mediated current by TEA (20 mM) and GSK-7975A (5 μM) in BSO-pretreated cells. On the left, traces of currents recorded in BSO-treated cells with TEA (20 mM) or GSK 7579A are shown. On the right, recapitulative histograms plot P_2_Y receptor-mediated current densities measured at +50 mV in BSO-pretreated cells (*n* = 3 for each condition). *p < 0.05 vs. BSO-treated cells without any blocker (ANOVA followed by Holm-Sidak *post-hoc* test).

To further characterize membrane conductance changes elicited by [GSH]_i_ manipulations on P_2_Y receptor signaling, electrophysiological recordings were carried out. The application of ADP resulted in the occurrence of a potassium current reversing around −77.0 ± 3.7 mV (*n* = 4) which exhibited an inward rectification at high voltages and a high sensitivity to 20 mM tetraethyl ammonium (TEA, Figure 7A). In cells pretreated with SFN, the current density measured at + 50 mV was not significantly decreased from 6.61 ± 1.94 pA/pF (*n* = 4) in control cells to 2.54 ± 0.66 pA/pF (*n* = 5). Under these conditions, the reversal potential was not significantly different from the one obtained in control cells (−69.6 ± 27.4 mV, *n* = 5, Figure 7B). By contrast, in cells pretreated with BSO, the ADP-elicited current was dramatically and significantly increased as current density reached 33.2 ± 5.0 pA/pF (*n* = 3) at +50 mV with shifted reversal potential to −48.0 ± 2.9 mV (*n* = 3, Figure 7B). These results agreed with the sensitization of the P_2_Y receptor response as previously observed monitoring [Ca^2+^] changes (Figures 4A,B). Interestingly in BSO-treated cells, ADP-elicited currents were significantly reduced by 20 mM TEA as well as by 5 μM GSK-7975A. Indeed, in BSO-treated cells the current density at +50 mV was 7.7 ± 4.4 pA/pF (*n* = 3) and 3.9 ± 1.8 pA/pF (*n* = 3), in the presence of TEA and GSK-7975A, respectively. Under these conditions, both TEA and GSK shifted the reversal potential from −48.0 ± 2.9 mV (*n* = 3) to −35.94 ± 0.05 mV (*n* = 3) and −27.5 ± 1.2 mV (*n* = 3) respectively (Figure 7C).

## Discussion

**FIGURE 8:**
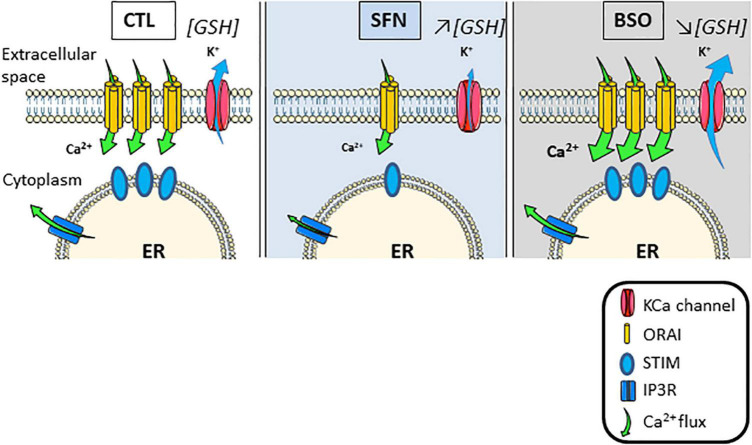
Scheme summarizing the adaptations of store-operated Ca^2+^ entry triggered by IP3 and initiated by [GSH]_i_ changes.

We show here that modulating cell redox status by manipulating GSH metabolism primarily affects the IP_3_/Ca^2+^ pathway in C6 astroglioma cell line (see scheme in Figure 8). Firstly, GSH depletion with BSO greatly sensitizes this pathway as evidenced by the unveiling of SOCE after the stimulation of G_*q*_-linked P_2_Y receptors. This increase in SOCE most likely results from the sensitization of CRAC as evidenced by the inhibitory action of CRAC selective blocker, i.e., GSK-7975A. CRAC sensitivity to redox status most likely results from the presence of cysteine residues occurring in the structure of Orai proteins which constitute CRAC (Bogeski et al., 2010) but also in STIM proteins structure (Gibhardt et al., 2020). However, under oxidative conditions, a suppression of SOCE is observed because of STIM2 (Gibhardt et al., 2020) or Orai (Bogeski et al., 2010) oxidization. Therefore, enhanced SOCE observed here could result from a rise in CRAC trafficking at the membrane as evidenced by the increased potency of CRAC blocker, although, this treatment does not appear to result from changes in STIM and Orai proteins expression *via* genomic actions. By contrast, SFN treatment leads to a desensitization of the IP_3_/Ca^2+^ entry which could be attributed to genomic actions. Indeed, the fact that SFN treatment does not affect either basal Ca^2+^ or Ca^2+^ load in the ER or SOCE mediated by SERCA inhibition further excludes changes in [Ca^2+^]_i_ handling mediated by SFN. However, previous data raised on rabbit gastric mucosal cells have demonstrated that Ca^2+^ release from the ER was reduced when GSH content was increased (Wong and Tepperman, 1994). As we observe that STIM2, Orai1, and Orai3 proteins expressions are reduced by this treatment, reduced coupling between STIM2 and Orai more likely explains the reduced Ca^2+^ entry after P_2_Y receptors activation than an inhibition of Ca^2+^ release from the ER. In support of this hypothesis, diminished expression of STIM and Orai proteins has been shown to be at the origin of decreased SOCE and increased resistance to oxidative damage in HT22 cells (Henke et al., 2013). On the opposite, increased SOCE is a hallmark of increased sensitivity to oxidative stress in neuronal cells (Crouzin et al., 2007; Henke et al., 2013) and SOCE inhibition protects neuronal cells from oxidative stress-induced apoptosis (Rao et al., 2013).

Surprisingly, SOCE elicited by SERCA blockade with cyclopiazonic acid is rather insensitive to both BSO- and SFN-pretreatment. This could be linked to the fact that SERCA inhibition leads to great amounts of Ca^2+^ released from the ER which may overwhelm redox-mediated changes of CRAC (Kraft, 2015). Indeed, previous works have shown that STIM2 activation was more likely associated with moderate ER Ca^2+^ mobilization, while STIM1 could be recruited by strong ER Ca^2+^ depletion (Thiel et al., 2013). Interestingly, we observe that STIM1 gene protein expression remains constant independently of the treatment, while STIM2 gene and protein expressions are reduced by SFN pretreatment. These data could explain why no changes in SOCE induced by SERCA inhibition could be evidenced, especially in SFN-treated cells. Moreover, this reinforces the fact that GSH depletion modulates only physiological relevant stimuli as P_2_Y receptors activation and oxidative stress.

We have also tested whether redox-sensitive TRP channels including TRPC1, TRPM7 and TRPV1 gene expression in C6 was changed by [GSH]_I_ manipulations as they can produce cell adaptations to oxidative environment (Sakaguchi and Mori, 2020). We had previously shown that TRPV1 channels supported both cell sensitivity to oxidative stress and glutamate-induced SOCE in cultured neurons (Crouzin et al., 2010). In SFN-treated cells, TRPV1 gene expression was significantly enhanced. However, the direct activation of TRPV1 channels with capsaicin did not elicit any Ca^2+^ rise in these cells whatever the treatment. In addition, TRPA1 gene was inconsistently detected in C6 cells. The application of TRPA1 agonist JT010 (10 μM) did not elicit any changes in [Ca^2+^]_i_.

SFN “anti-aging” effect is known to involve the activation of the Nrf2 pathway and the stimulation of ARE DNA sequences and subsequent expression of antioxidant enzymes. Our data suggest that Nrf2 activation could also modulate SOCE-associated proteins gene expression leading to changes in Orai proteins both sensitive and insensitive to oxidization. Indeed, Orai3 protein, in comparison to Orai1, lacks the homolog of cysteine 195 residue which is reactive to oxidation (Bogeski et al., 2012). In this line, SFN has been demonstrated to activate genomic pathways independently of the Nrf2/ARE pathway leading to cell protection. Indeed, while the activation this pathway may repress the expression of many proteins involved in neuronal degeneration such as BACE (Bahn et al., 2019), SFN may trigger various intracellular pathways independently of Nrf2/ARE, including epigenetic mechanisms (Santín-Márquez et al., 2019), resulting in the reduced expression of proinflammatory proteins (An et al., 2016; Park et al., 2017). Such actions could explain the discrepancy observed here between SFN stimulatory action on mRNA expression and its concomitant repression of protein expression.

Astrocytes undergo complex changes during aging and neurodegenerative diseases. Indeed, in one hand, their activation is involved in neuroinflammation which endanger neurons *via* cytokines overproduction and on the other hand, atrophy and loss of activity leads to reduced neuronal protection (Rodríguez-Arellano et al., 2016; Verkhratsky, 2019). In this line, normal aging by itself appears to trigger astrocyte activation (Clarke et al., 2018). Studies performed on cultured astrocytes have shown that “old” astrocytes become more sensitive to oxidative stress elicited by tBuOOH and exhibit changes in intracellular calcium homeostasis as purinergic responses are magnified (Lin et al., 2007). These data are in good agreement with the data that we have obtained on BSO-treated cells. Moreover, Lin et al. (2007) had observed that old astrocytes are more prone to exhibit Ca^2+^ oscillations than young ones under purinergic stimulation. These oscillations may involve a cooperation between K_*Ca*_ potassium channels and CRAC. Astrocytes express K_*Ca*_ channels mediating Ca^2+^-dependent potassium currents (Verkhratsky and Nedergaard, 2018). These channels may co-localize with CRAC in many cell types (Guéguinou et al., 2014), including many cancer cell types where proliferation has been shown to be supported K_*Ca*_3.1 channel activity (Klumpp et al., 2017). Moreover, the interaction between K_*Ca*_3.1 and CRAC regulates [Ca^2+^]_i_ oscillations in glioblastoma (Catacuzzeno and Franciolini, 2018). We find here that the stimulation of P_2_Y receptors elicited K_*Ca*_ currents which were magnified by BSO treatment and sensitive to CRAC blocker GSK-7975A. Interestingly, such a dependence of K_*Ca*_3.1 on CRAC activation has also been evidenced in human mast cells. This mechanism seems to be required for IgE-mediated release of histamine from these cells (Duffy et al., 2015). One may thus speculate, that such an interaction could also play a critical role in the release of cytokines from astrocytes activated under oxidative stress conditions, as evidenced in several glial cell types (Lee et al., 2010). It could also account for dysfunction in Ca^2+^ homeostasis observed during aging (Lin et al., 2007) and under pathological conditions involving oxidative stress as Huntington’s disease (Jiang et al., 2016) and other models of neurodegenerative diseases (Shigetomi et al., 2019).

Although C6 astroglioma cells recapitulates most of the features of astrocytes and represent a suitable model to evaluate oxidative stress-induced toxicity and resistance, these cells are deriving from tumors and they could thus acquire specific phenotype with regard to oxidative stress handling. Indeed, cancer cells have developed metabolic strategies to prevent detrimental actions of ROS on themselves while they produce ROS in large amounts as oxidative stress required for tissue invasion. In this line, the overactivity of Nrf2 pathway appears to be a hallmark of cancer cells (Sporn and Liby, 2013; Rojo de la Vega et al., 2018). Therefore, compared to native astrocytes, one could expect that C6 cells exhibit increased resistance to oxidative stress. We may thus have underestimated the sensitive threshold to ROS in these cells. Further experiments should be performed on *in situ* astrocytes to evaluate their sensitivity to ROS with aging.

## Data Availability Statement

The original contributions presented in the study are included in the article/supplementary material, further inquiries can be directed to the corresponding author/s.

## Author Contributions

NM, YS, TC, JG, CC-S, JR, and MV performed the experiments. NM, TC, JG, PC, J-BT, M-CJ, AB, MR, CM, JR, and MV designed the experiments and analyzed the data. All authors contributed to the article and approved the submitted version.

## Conflict of Interest

The authors declare that the research was conducted in the absence of any commercial or financial relationships that could be construed as a potential conflict of interest.

## Publisher’s Note

All claims expressed in this article are solely those of the authors and do not necessarily represent those of their affiliated organizations, or those of the publisher, the editors and the reviewers. Any product that may be evaluated in this article, or claim that may be made by its manufacturer, is not guaranteed or endorsed by the publisher.
